# Serum Galectin-3 level, not Galectin-1, is associated with the clinical feature and outcome in patients with acute ischemic stroke

**DOI:** 10.18632/oncotarget.18211

**Published:** 2017-05-25

**Authors:** Han Dong, Zhi-Hao Wang, Na Zhang, Si-Da Liu, Jian-Jun Zhao, Song-Yan Liu

**Affiliations:** ^1^ Department of Geriatric Medicine, The First Affiliated Hospital of Jilin University, Changchun, China; ^2^ Department of Electrical Diagnosis, The General Hospital of Fourth Hospital of Jilin University, Changchun, China; ^3^ Department of Thoracic Surgery, Xinhua Hospital Affiliated to Shanghai Jiao Tong University School of Medicine, Shanghai, China; ^4^ Department of Neurology, The Affiliated Hospital to Changchun University of Chinese Medicine, Changchun, China; ^5^ Department of Neurology, China-Japan Union Hospital of Jilin University, Changchun, China

**Keywords:** ischemic stroke, Galectin-3, prognosis

## Abstract

**Aim:**

To study the diagnostic and prognostic role of serum galectin-1 (Gal-1) and -3 (Gal-3) in acute ischemic stroke (AIS) patients.

**Methods:**

We enrolled 233 patients with first-ever acute ischemic stroke and 252 healthy controls in this study. The AIS severity was evaluated by National Institutes of Health Stroke Scale (NIHSS) scores. The serum Gal-1 and -3 levels were determined. All patients were followed for 1 years and the functional outcome were evaluated by modified Rankin Scale (mRS) scores.

**Results:**

We found that AIS patients had higher serum Gal-1 and -3 levels than controls. The serum Gal-3 level was closely associated with the AIS severity indicated by NHSS and infarction volume. Serum Gal-3 levels were significantly higher in patients with a poor outcome indicated by mRS scores than those in patients with a good outcome. In contrast, the serum Gal-1 is not associated with the severity and outcome of acute AIS patients. Our in vitro studies show that Gal-3 knockdown with siRNA dramatically increased the culture neuron cell viability and reduced apoptosis under oxygen glucose deprivation treatment. Meanwhile, the pro-inflammatory cytokine expression decreased with the inhibition of Gal-3.

**Conclusion:**

Our finding provides a novel biological marker, serum Gal-3, for monitor of acute AIS patients.

## INTRODUCTION

Acute ischemic stroke (AIS) is among the most common public health concerns worldwide since it is a major cause of long term disability and death [1] [2]. The prediction of outcome subsequent to AIS has been an area of interest for neuroscientists. Previous studies suggested that inflammation and angiogenesis are key factors in the pathological response of ischemic stroke [3] [4] [5].

Galectins (Gals) are a family of β-galactoside-binding lectins characterized by their binding affinity for β-galactosides [6] [7]. Gals are produced by a variety of vascular, interstitial, epithelial, and immune cells [8]. Fifteen members of this family have been identified to date [9]. Gal-1 and Gal-3 are two members mostly studied. These two galectins participate in a variety of biological processes, including cell proliferation, angiogenesis and inflammation regulation [10] [11] [12]. Gal-1 and -3, have been also implicated in a variety of central nervous system disorders including gliomas, spine cord injury and neuroinflammation [13] [14] [15] [6]. Recent study reports that Gal-1 improves functional outcome in rats following cerebral ischemia [16]. Increased Gal-3 was also observed in microglia during neonatal hypoxic-ischemic brain injury and contributes to hypoxic-ischemic brain injury [17]. Gal-3 mediates post-ischemic tissue remodeling and exogenous Gal-3 increases proliferation of endothelial and neural progenitor cells, and enhances microvessel density in ischemic rat brain [18].

However, the association between Gal-1 and -3 and acute ischemic stroke in a clinical setting remain unknown. In this study, we enrolled patients with AIS and measured their serum Gal-1 and -3 levels. We found that serum Gal-3 at admission was closely associated with the clinical futures and outcome of theses patients, suggesting that serum Gal-3 may be used as a biological marker to predict the prognosis of AIS.

## MATERIALS AND METHODS

### Patients and study design

From February 2010 to September 2013, we enrolled 233 patients with first-ever acute ischemic stroke from our hospital. All patients were irrelevant Han Chinese and were admitted to our department within 24 hours of the new onset of focal or global neurological event. All patients had brain imaging (either CT or MRI) within 24 hours after admission. The diagnosis of acute ischemic stroke was according to the World Health Organization criteria [19]. We excluded patients with hemorrhagic stroke, recurrent IS, cancer, acute or chronic inflammatory disease. Stroke subtype was classified according to TOAST (Trial of Org 10172 in Acute Stroke Treatment) criteria [20].

Stroke severity at admission was assessed on admission using the National Institutes of Health Stroke Scale (NIHSS) by two neurologists who were unaware of the study protocol. The NIHSS score ranges from 0 to 34 and higher values reflect more severe neurological damage. MRI with diffusion-weighted imaging was performed to determine the infarct volume as previously described [20]. The baseline demographic and clinic information (such as age, sex, hypertension, lipid profile, smoking status, and family history et al) were obtained from patient’s medical records. All patients received standard therapy during hospitalization. The functional outcome assessed by the modified Rankin Scale (mRS) 12 months after discharged from hospital. A favorable functional outcome was defined as a mRS of 0-2 points, whereas an poor outcome was defined as a mRS of 3-6 points [20] .

We also enrolled 252 healthy volunteers matched for age and gender as normal controls. All these controls receive regular medical checkup and are free of any disease.

A written consent was obtained from all patients before enrollment in the study, and the Ethical Committee of our hospital approved the protocol, which was in accordance with the ethical guidelines of the 1975 Declaration of Helsinki.

### Blood collection and quantification

The peripheral blood samples were obtained by venous puncture immediately after the diagnosis was established (within 12 hours after admission). Serum samples were stored at −80 °C before being analyzed. All samples were thawed only once prior to use. Gal-1 and Gal-3 levels were measured using commercially available enzyme-linked immunosorbent assay (ELISA) kits (R&D Systems, Minneapolis, MN, USA; Catalogue number DGAL10, DGAL30, DGAL90, and DGBP30B). According to the instructions, the intra-assay and inter-assay coefficients of variation are, respectively, 5.7-8.8% and 7.5-9.5% for Gal-1, 3.0-4.4% and 6.8-8.6% for Gal-3.

### Quantitative analysis of the relative amount of Gal-1 and Gal 3 mRNA by quantitative real-time PCR (q-PCR)

The nucleic acid extraction was performed from blood samples according to the protocol accompanying the reagent TRIzol (Invitrogen). The relative quantification q-PCR assay for Gal-1 and Gal 3 mRNA expression was performed in an ABI Prism 7300 Sequence Detector System (Applied Biosystems, Foster City, CA, USA), according to the instructions for the SYBR Green PCR Core Reagent (Applied Biosystems). The q-PCR assays were performed in a total volume of 50 μL, containing 10 μL of SYBR Green Master Mix (Applied Biosystems), 25 ng of cDNA, and 0.4 μM of ANXA1 and 0.5 μM LGALS1 primers. The fluorescence signal was measured in the extension phase of the PCR reaction, and a threshold value (CT) of fluorescence in the exponential part of the amplification curve was selected. The primers for Gal-1 are: F5′-TGCAACAGCAAGGACGGC-3′; R: 5′-CACCTCTGCAACACTTCCA-3′; for Gal-3: F5′-CAGAATTGCTTTAGATTTCCAA-3′; R5′-TTATCCAGCTTTGTATTGCAA.

### si-RNA against Gal-1 and Gal-3 in human cortical neuronal (HCN) cell lines culture and oxygen glucose deprivation

Human brain cortical cells (HCN-1A, ATCC CRL-10442) were cultured in Eagle’s minimal essential medium (MEM) with 2 mM l-glutamine and 1.0 mM sodium pyruvate supplemented with 10% heat-inactivated fetal bovine serum. Cells were grown to confluence at 37°C in 5% CO2. Three types of galectin-3 siRNA duplex (type I, 5- UCCAGACCCAGAUAACGCAUCAUGG-3; type II, 5-UAAAGUGGAAGGCAACAUCAUUCCC-3; and type III, 5-AUAUGAAGCACUGGUGAGGUCUAUG-3) and a scramble RNAi as a negative control. Two types of sequences for Gal-1 siRNA was 5’-UUGCUGUUGCACACGAUGGUGUUGG-3’and5’-GCUGCCAGAUGGAUACGAAUUTGGA-3’. All sequences were purchased from Invitrogen Life Technologies (Carlsbad, CA, USA).

After siRNA transfection, neurons were treated with oxygen glucose deprivation (OGD), 1 h in an environment containing 1% oxygen and glucose free media.

### Determination of cell viability

Cell viability was measured by 3-(4,5-dimethylthiazol-2-yl)-2,5-diphenyltetrazolium bromide (MTT) colorimetric assay. Briefly, the neuron cells were placed in 96-well plates at a final concentration of 1 × 105 per mL in culture medium, and were incubated at 37°C with 5% CO2. After Gal-1 and 3 si-RNA transfection, 10 μl MTT was added to each well, and incubated at 37°C for another 2 h. The absorbance at 595 nm was read on a microplate reader.

### TUNEL assay

Terminal deoxynucleotidyl transferase-mediated uridine 5′-triphosphate-biotin nick end labeling (TUNEL) staining was performed to detect the apoptosis in cultured cells receiving Gal-1 and 3 si-RNA. The apoptosis rate was calculated by dividing the TUNEL positive cell number by total cell numbers per field.

### Western blotting analysis

Proteins were extracted from HCN cells and the protein concentrations were assessed using a BCA assay kit (TaKaRa BIO INC, Japan). The protein samples were resolved in a 10-12% sodium dodecyl sulfate (SDS)-polyacrylamide gel electrophoresis. Proteins were then transferred to polyvinylidene fluoride (PVDF) membrane, and blocked with 5% nonfat milk in Trisbuffered saline-Tween (TBST) 20 for 2 h at room temperature. Membranes were then incubated with primary antibody overnight. The antibodies were shown as follows: anti-Caspase-3, anti-Bcl2, anti-IL1, anti-IL6 and anti-NF-kb (1:1000; Santa Cruz Biotechnology,USA). An anti-GAPDH antibody was used as a loading control. Membranes were washed and incubated for 2 h in the presence of appropriate horseradish peroxidase (HRP)-conjugated secondary antibody. The positive reaction was visualized by using 3, 3’-diaminobenzitine (DAB) solution (Sigma, St. Louis, MO) with a chemiluminescent Immobilon Western blotting detection system.

### Statistical analysis

Results are expressed as percentages for categorical variables and as medians mean±standard error for the continuous variables. Correlations were determined using Spearman correlation analyses. Multivariate analysis was performed by binary logistic regression analysis with adjustment for conventional confounding factors. The adjusted OR (odds ratios) with the corresponding 95% CIs (confidence intervals) were calculated. Receiver operating characteristic (ROC) curves were utilized to evaluate the accuracy of Gal-1 and -3 to discriminate stroke patient from controls and to predict the good and poor outcomes. Area under the curve (AUC) was acquired. Two-sided P values of less than 0.05 were regarded as significant. All statistical analysis was performed with SPSS for Windows, version 16.0 (SPSS Inc., Chicago, IL, USA).

## RESULTS

### Baseline characteristics of study samples

The baseline characteristics of AIS patients and controls are described in Table 1. There is no significant difference in age, sex distribution, BMI, DBP, serum glucose, serum Cr, serum TC, TG and HDL between two groups. However, stroke patients had more smoker percentage and higher serum LDL levels than controls. Notably, the stroke patients had significantly higher Gal-1 and -3 mRNA expression and serum protein levels than controls (all *P* < 0.001, Table 1).

**Table 1:** The baseline characteristics of IS patients and controls.

Variables	Stroke patients (n=233)	Control (n=252)	*P* value
**Age (years)**	55.5±6.3	55.3±8.8	NS
**Sex (Male,%)**	166 (71.2%)	177 (70.3%)	NS
**BMI (k/m2)**	55.5±6.5	55.3±8.10	NS
**IS family history**	45 (19.3%)	46 (18.2%)	NS
**Smoking status**			
never smoker	67 (28.8%)	78 (31.0%)	NS
ever smoker	85 (36.5%)	89 (35.3%)	
current smoker	81 (34.7%)	85 (33.7%)	
**SBP (mmHg)**	143.7±16.6	124.3±12.8	<0.001
**DBP (mmHg)**	80.5±6.3	79.3±9.4	NS
**TC** **(mmol/L)**	4.7±1.1	4.7±1.0	NS
**TG (mmol/L)**	1.7±1.0	1.7±1.2	NS
**HDL (mmol/L)**	2.1±0.6	2.0±0.7	NS
**LDL (mmol/L)**	2.7±0.9	2.2±0.8	<0.001
**Hs-CRP (mmol/l)**	2.6±0.8	2.2±0.6	<0.001
**s-Cr** **(mmol/l)**	56.8±6.5	55.9±7.4	NS
**Gal-1 (pg/ml)**	48.5±20.4	43.9±22.5	<0.001
**Gal-3 (pg/ml)**	43.5±9.9	25.2±9.9	<0.001
**Gal-1 (relative expression)**	2.5±0.4	1.7±0.3	<0.001
**Gal-3 (relative expression)**	2.7±0.2	1.6±0.2	<0.001
**Stroke symptom n (%)**			
total anterior circulation syndrome	38 (16.3%)	-	
lacunar syndrome	87 (37.3%)	-	
partial anterior circulation syndrome	54 (23.2%)	-	
posterior circulation syndrome	54 (23.2%)	-	
**Stroke etiology n (%)**			
Small-vessel occlusive	54 (23.1)	-	
Large-vessel occlusive	50 (21.5)	-	
Cardioembolic	98 (42.1)	-	
Other	18 (7.7)	-	
Unknown	13 (5.6)	-	

Abbreviations: IS, ischemic stroke; BMI, Body mass index; HDL, High-density lipoproteins; LDL, Low-density lipoproteins; BP, systolic blood pressure; BP, diastolic blood pressure

**Figure 1:**
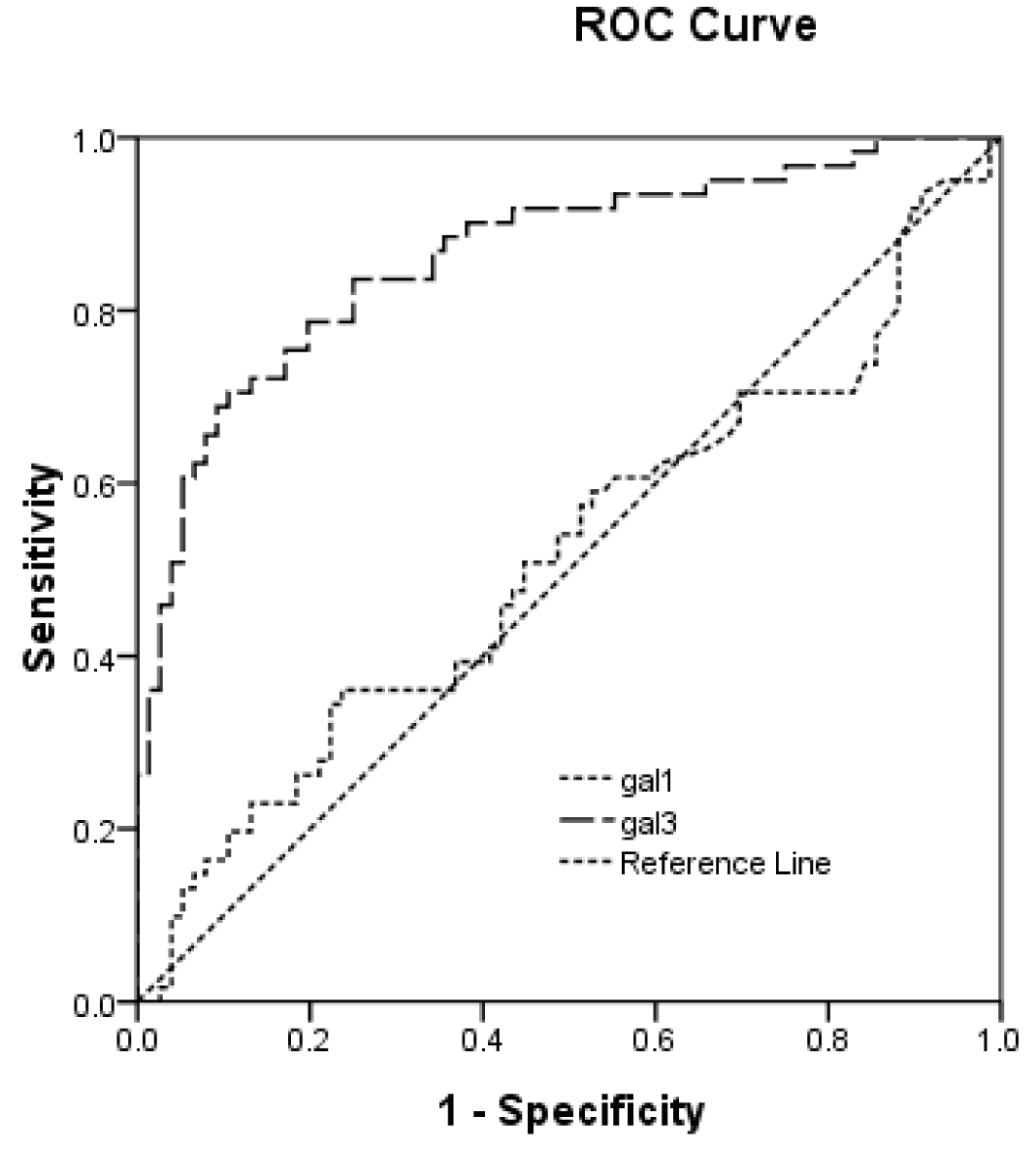
The ROC curves of Gal-1 and -3 in determining ischemic stroke patients from controls The ROC analyses of Gal-3 and Gal-3 in discriminating stroke from controls. Gal-3 serum level can distinguish stroke patients from healthy controls (AUC = 0.884, 95%: 0.827-0.927, *P* < 0.001). At the cut-off value of 53.5 pg/ml, the specificity for stroke prediction was 89.3% and sensitivity was 75.8%. In contrast, the serum Gal-3 levels did not distinguish stroke patients from controls (AUC = 0.552, 95% CI: 0.455-0.648, *P* = 0.3).

We performed the ROC analyses to test if serum Gal-1 and Gal-3 can discriminate AIS from controls. Our data show that Gal-3 serum level had diagnostic value for stroke (AUC = 0.884, 95%: 0.827-0.927, *P* < 0.001). At the cut-off value of 53.5 pg/ml, the specificity for AIS prediction was 89.3% and sensitivity was 75.8%. In contrast, the serum Gal-1 levels did not distinguish stroke patients from controls (AUC = 0.552, 95% CI: 0.455-1.048, *P* = 0.3) (Figure 1). However, the Gal-1 and -3 mRNA did not show the ability to discriminate AIS from controls (Gal-1 mRNA, AUC = 0.543, 95% CI: 0.455-0.648, *P* = 0.3; Gal-3 mRNA, (AUC = 0.552, 95% CI: 0.455-0.648, *P* = 0.3).

We also performed the multiple regression analyses to determine the independent risk factor for AIS in this study. Our data show that only serum Gal-3 predicts the stroke incidence (OR = 1.656, 95%CI: 0.132-2.312, *P* < 0.001) with adjustment of conventional clinical factors, including age, sex, BMI, blood pressure levels, TC, TC,HDL, LDL, serum Cr, serum hs-CRP et al. Although the serum Gal-1 levels were higher in stroke patient, it is not an independent factor associated with stroke in our study (OR = 1.001, 95%CI: 0.937-1.365, *P* = 0.064).

We next compared the association between serum Gal-1 and Gal-3 levels and stroke characteristics. We found that serum Gal-3 level increased with the severity of stroke defined by the NIHSS and mRS scores at admission. Pearson correlation analyses reveal serum Gal-3 level was highly correlated with both the NIHSS and mRS scores of patients (r  =  0.871, *P* < 0.0001; Gal-1: r = 0.728 *P* < 0.001, respectively). However, serum Gal-1 level and its mRNA did not show this correlation with NIHSS score (r = 0.127, *P* = 0.076).

**Figure 2:**
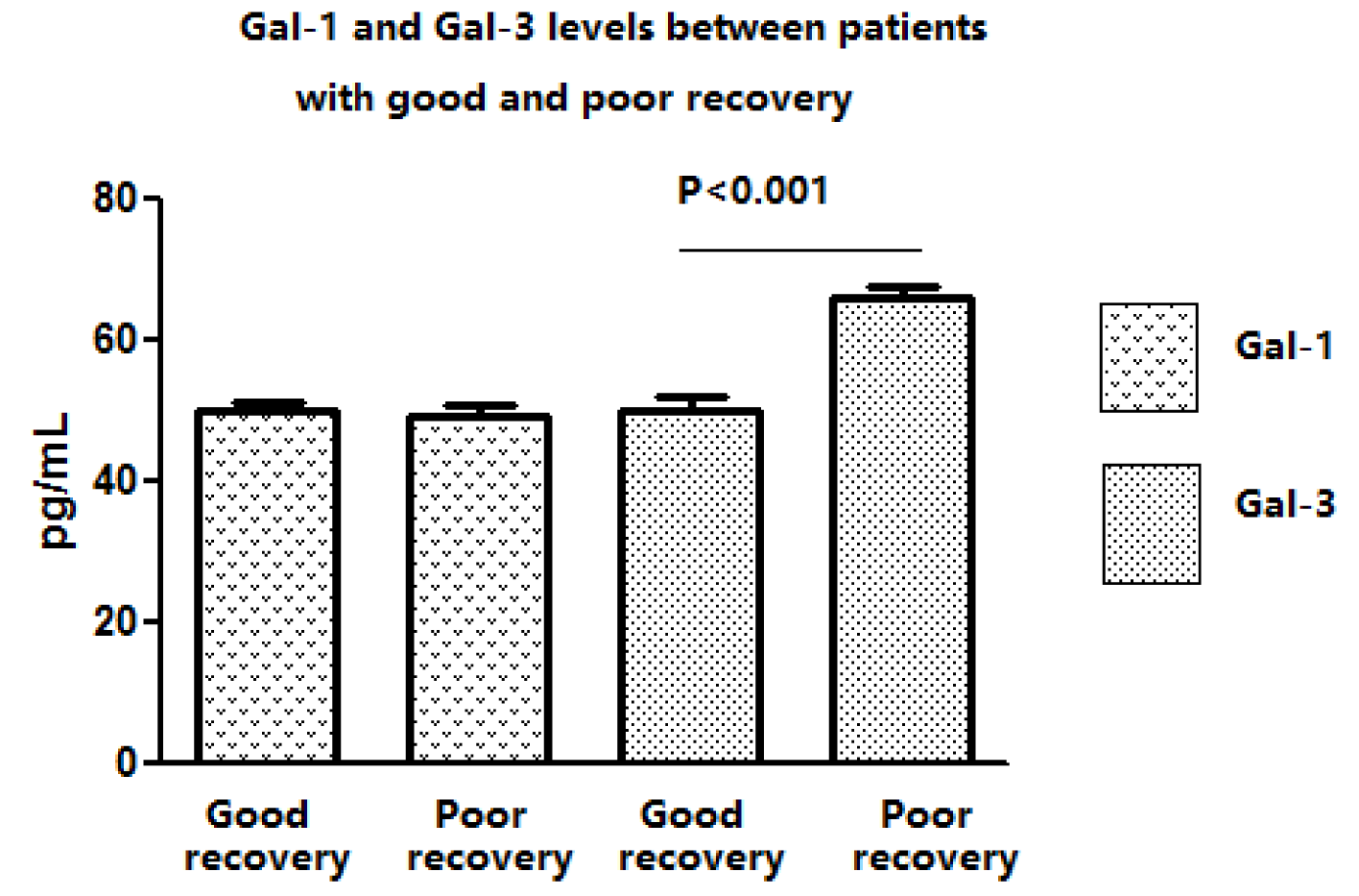
The serum Gal-1 and -3 levels in IS patients with a poor and good outcome Our data reveal that serum Gal-3 levels were significantly higher in patients with a poor outcome than those in patients with a good outcome (49.6±1.2 vs. 60.1±0.66, pg/ml, *P* < 0.001). Gal-1 serum level was similar between patients with good and bad recoveries (51.4±2.1 vs.51.2±2.6, pg/ml, *P* = 0.941, Figure 2).

There were 183 patents completed follow-up study. There were 6 patients died and 44 were lost during the follow-up period. Based on the mRS score one year after discharge from our hospital, there are 113 patient gain good recoveries (mRS score: 0-2 points) while 70 had bad recovery (mRS score: 3-6 points). We found that serum Gal-3 levels were significantly higher in patients with a poor outcome than those in patients with a good outcome (49.6±1.2 vs. 60.1±0.66, *P* < 0.001). Gal-1 serum level was similar between patients with good and bad recoveries (51.4±2.1 vs.51.2±2.6, *P* = 0.941, Figure 2).

**Figure 3:**
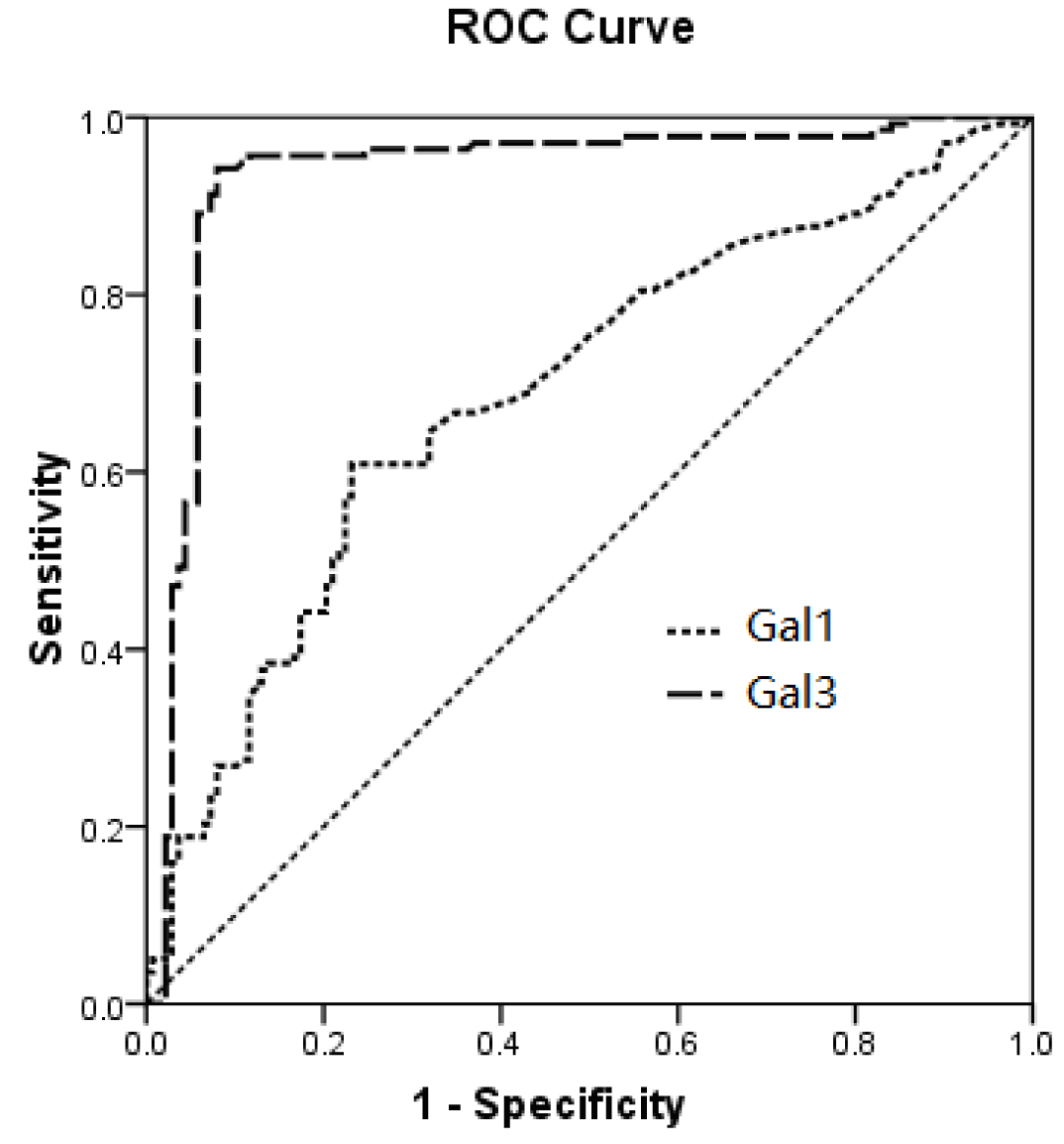
The ROC analyses of Gal-1 and -3 in distinguish patients with good recovery from those with bad recovery We found that Gal-3 serum level was able to predict the functional outcome of IS patients (AUC = 0.884, 95CI:0.827-0.941, *P* < 0.001) with sensitivity of 88.4% and specificity of 76.9% at the cut-off value of 53.5. The Gal-3 had no predictive value on the outcome of IS patients.

We further performed ROC analyses to see the ability of Gal-1 and -3 in distinguish patients with good recovery from those with bad recovery. We found that Gal-3 serum level was able to predict the functional outcome of AIS patients (AUC = 0.884, 95CI:0.827-0.941, *P* < 0.001) with sensitivity of 88.4% and specificity of 76.9% at the cut-off value of 53.5 as shown in Figure 3. The Gal-1 had no predictive value on the outcome of AIS patients.

**Figure 4:**
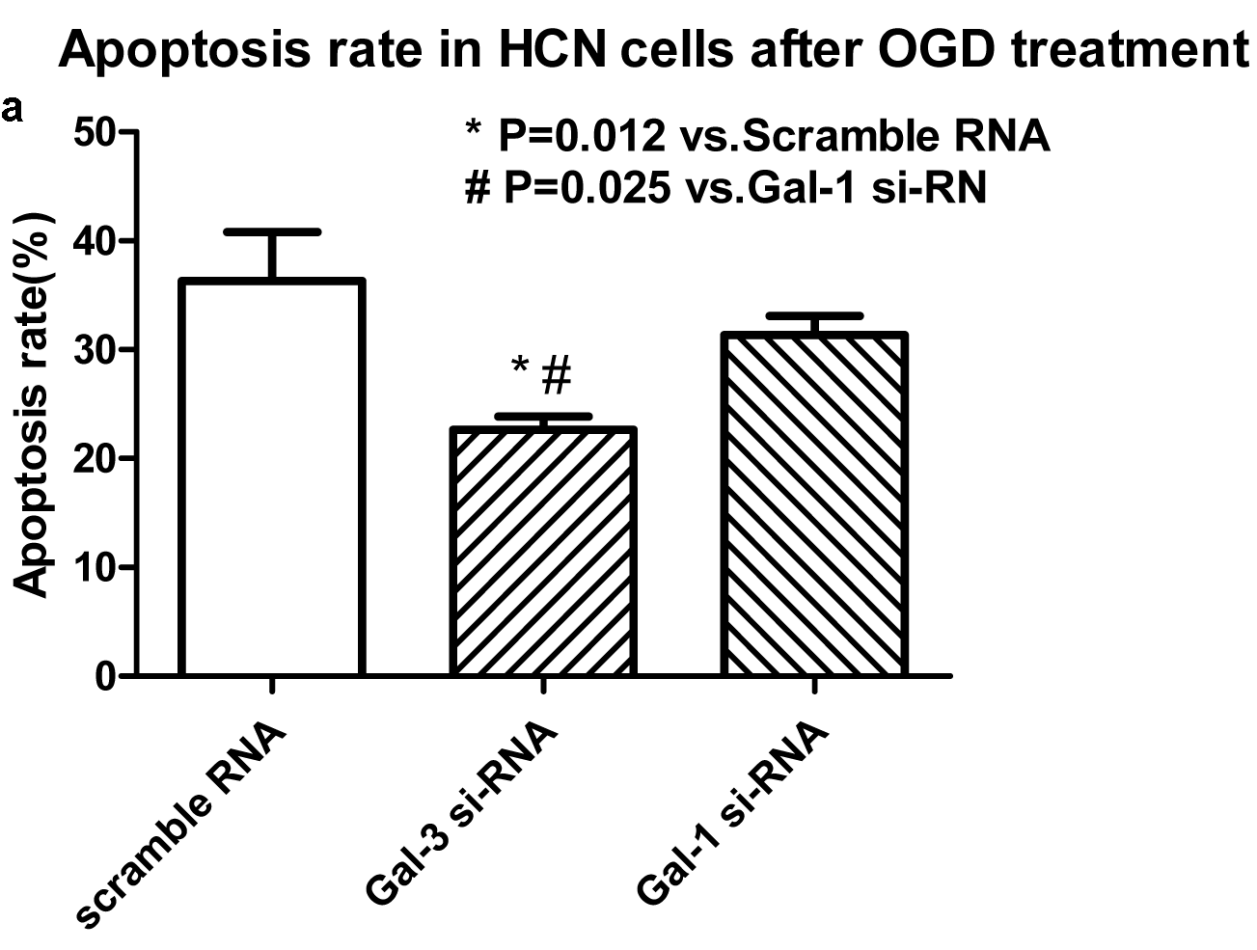

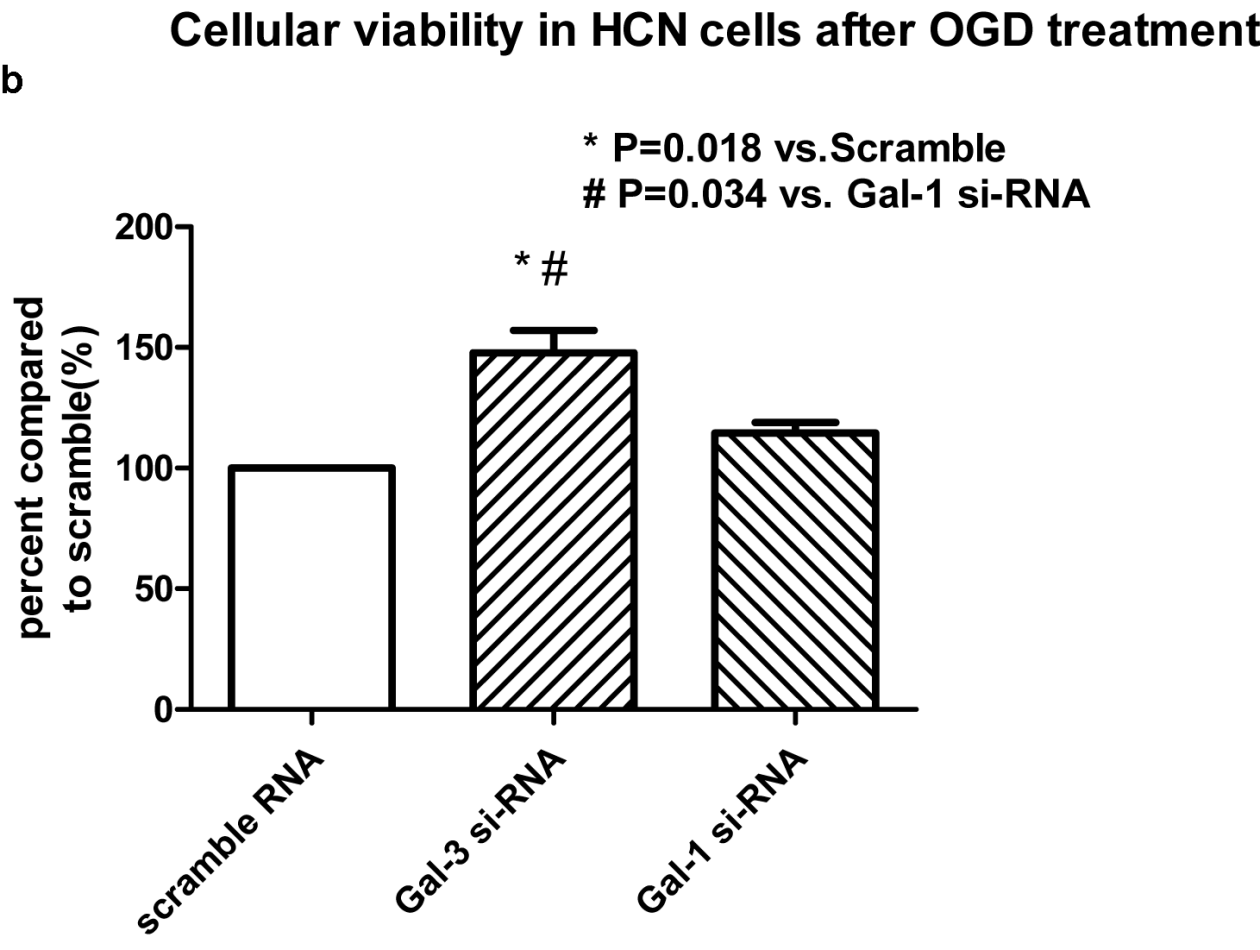
Gal-3 si-RNA transfection induced a significant reduction in apoptosis (**a**), but an increase in cell viability (**b**) compared to cells receiving scrambled RNA. Gal-1 si-RNA did not affect the cell apoptosis and viability.

To mimic the inflammatory stress HCN cells underwent during ischemic, we used OGD treatment after si-RNA transfection, followed by apoptosis and cell viability assays. In our *in vitro* study, we observed that after Gal-3 si-RNA transfection, there are a significantly reduction in apoptosis, but an increase in cell viability compared to cells receiving scramble RNA. These findings suggest that Gal-3 exerts a detrimental effect for cell fate (Figure 4a and 4b). The Gal-1 si-RNA transfection slightly affects the cell viability and apoptosis (Figure 4a and 4b), but far lower than Gal-3.

**Figure 5:**
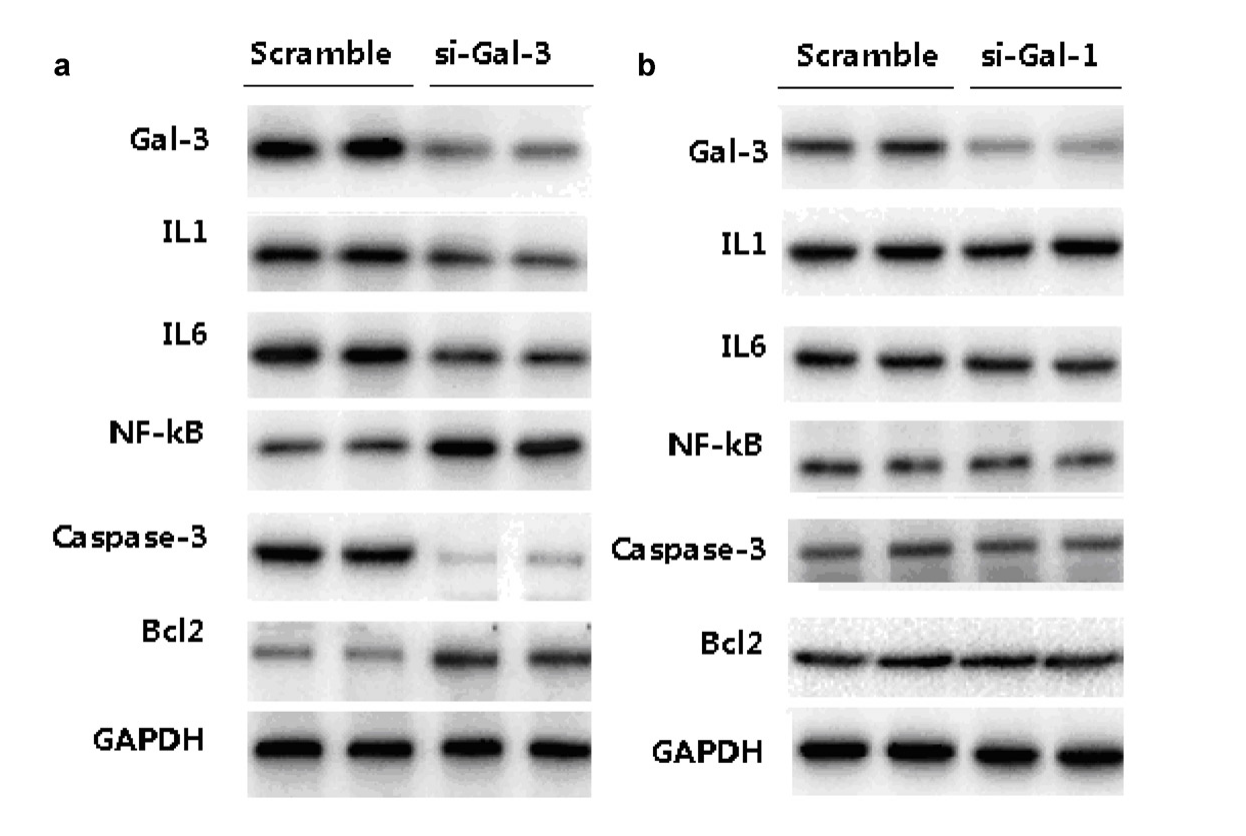
The expressions of pro-inflammatory cytokines and apoptosis-related proteins after si-Ga3 (**a**) and si-Gal-1 (**b**) transfection.

Meanwhile, our western blot data show that Gal-3 si-RNA markedly reduced the protein levels of pro-inflammatory cytokines, including IL1, IL6 and NF-kb as well as apoptosis related proteins, such as Casepase3, but increases an anti-apoptoic protein Bcl2 level. Gal-1 did not markedly modify these protein levels in cells (Figure 5).

## DISCUSSION

In this study, we found that serum Gal-3 was closely associated with the clinical feature of AIS (including MHSS and focal volume) and also predict the functional outcome 12 month after discharge from hospital. Our finding provide a novel biological marker for clinical monitor of AIS patients.

Ischemic stroke usually initiates inflammation and oxidative/nitrosative stress leading to neuronal death [5] [21] [22]. Localized cerebral ischemia is coupled with focal inflammatory reactions that ultimately end with cell death. Pro-inflammatory mediators and ROS are released by microglial cells following tissue injury induced by ischemia [23]. Previous researchers observed elevated inflammation biomarkers including white blood cell count, elevated neutrophils ratio, and elevated erythrocyte sedimentation rate were associated with short-term clinical outcomes [24]. Several serum inflammation markers, such as procalcitonin, hs-CRP have been documented as independent predictor of long-term mortality after ischemic stroke in a Chinese sample [25].

Gal-3 is a member of a class of carbohydrate-binding proteins and plays a role in a number of cellular functions such as cell proliferation, angiogenesis and differentiation [26]. Growing evidence suggests that galectin-3 is involved in fine tuning of the inflammatory responses at central neurological system. Up-regulation of Gal-3 and IGF-1 in a subset of activated/proliferating microglial cells after stroke. Disruption of the galectin-3 gene significantly alters microglia activation and induces approximately 4-fold decrease in microglia proliferation [27].

Serum Gal-3 has been widely used as markers in a variety of disease including solid cancer [28][29][30]. In cardiovascular system, a study reported that serum galectin-3 concentrations were significantly higher in the coronary heart disease (CAD) patients than in the controls. Gal-3 levels were correlated positively with BMI, high-sensitivity C-reactive protein, the total number of diseased vessels, the number of plaques and the calcified plaque type. In addition, Gal-3 levels were found to be a significant independent predictor of coronary atherosclerosis in type 2 diabetic patients [31]. Circulating Gal-3 was highly associated with renal function in outpatients with heart failure (HF) [32]. Higher concentration of Gal-3 is also being viewed as a marker of cardiac fibrosis, which is associated with increased risk for incident HF and mortality in patients [33].

Gal-3 expression has been identified as a useful tool in the differential diagnosis of posterior fossa tumors in children. All pilocytic astrocytomas patients and ependymomas strongly showed Gal-3 expression, whereas no immunostaining was observed in medulloblastomas and diffuse astrocytomas [34]. Another study reported that Galectin-3 as an immunohistochemical tool to distinguish pilocytic astrocytomas from diffuse astrocytomas, and glioblastomas from anaplastic oligodendrogliomas [35].

Gal-3 is also involved in the repair response after brain ischemia. An up-regulated expression of Gal-3 in the ischemic brain following transient middle cerebral artery occlusion was observed in rats. *In vitro*, Gal-3 treatment stimulated the proliferation of endothelial cells and neural progenitors. Inhibition of Gal-3 activity decreased ischemia-induced angiogenesis and the proliferation of neural progenitors, suggesting that Gal-3 expressed by activated microglia/infiltrating macrophages and astrocytes in the ischemic brain may play a role in post-ischemic tissue remodeling by enhancing angiogenesis and neurogenesis [18]. The role of Gal-3 in ischemic stroke in a clinical setting remains unknown. For the first time, we reported that Gal-3 serum level is associated with the disease severity and functional recovery. This finding expends our current knowledge of Gal-3 in ischemic stroke.

In order to explore the possible mechanism by which Gal-3 affect the outcome of stroke patients, we used OGD condition to mimic the inflammatory stress in cultured HCN cells underwent during ischemic. We found that Gal-3 knockdown by si-RNA transfection significantly reduced apoptosis, but increases cell viability compared to cells receiving scramble RNA under OGD condition. Meanwhile, our western blot data show that Gal-3 si-RNA markedly reduced the protein levels of pro-inflammatory cytokines, including IL1, IL6 and NF-kb as well as apoptosis related proteins, such as Casepase3, but increases an anti-apoptoic protein Bcl-2 level. Similar finding has bee reported in different cell types, e.g., downregulating Gal-3 via RNA interference inhibits proinflammatory cytokine production by human monocyte-derived dendritic cells. Silencing of Gal-3 also increase the expression of several pro-apoptotic genes and augments the sensitivity of gastric cancer cells to chemotherapeutic agents [36]. Another study reported that *in vivo* siRNA knockdown of Galectin-3 inhibited myofibroblast activation after hepatic injury [37]. However, it should be noted that Gal-3 leads to attenuation of apoptosis through Bax heterodimerization in human thyroid carcinoma cells [38]. This finding are consistent with previous studies by others showing the detrimental effect of Gal-3 on cell destine.

Several limitations should be address in this study. Firstly, the sample size is relatively small and only Chinese patients were included in this study. Thus the conclusion of this study needs to be further testified in future study with larger sample size and different ethical population. Secondly, we did not study the effect of Gal-3 on the angiogenesis and neuron cell survival, which are directly related to the clinical outcome of acute ischemic stroke patients. Thirdly, we did not have explanation why Gal-1 was not associated with the clinical feature and prognosis of ischemic stroke patients.

## Abbreviations

Gal: Galectin
IS: Ischemic stroke
NIHSS: National Institutes of Health Stroke Scale
mRS: Modified Rankin Scale
OR: Odds ratios
CI: Confidence intervals
ROC: Receiver operating characteristic
AUC: Area under the curve
